# Divergent climate-growth responses in radial growth of Chinese pine forests with varying health conditions in the semi-arid Loess Plateau, China

**DOI:** 10.3389/fpls.2026.1809811

**Published:** 2026-04-10

**Authors:** Li Hongyi, Li Xuchun, Yu Haixia, Zhang Qindi, Li Zongshan

**Affiliations:** 1 Soil and Water Conservation Scientific Research Institute of Dingxi City, Dingxi, Gansu, China; 2 College of Life Sciences, Shanxi Normal University, Taiyuan, China; 3 State Key Laboratory of Regional and Urban Ecology, Research Center for Eco-Environmental Sciences, Chinese Academy of Sciences, Beijing, China; 4 Shaanxi Yan’an Forest Ecosystem National Observation and Research Station, Beijing, China; 5 National Observation and Research Station of Earth Critical Zone on the Loess Plateau in Shaanxi, Xi’an, China

**Keywords:** Chinese pine plantations, drought stress, growth decline, semi-arid region, the Loess Plateau, tree rings

## Abstract

**Introduction:**

Global climate change–induced extreme drought has triggered widespread forest growth decline and tree mortality worldwide, making the processes of forest decline and their responses to environmental conditions a major research focus. In the Longtan Catchment of the Loess Plateau, Chinese pine (*Pinus tabulaeformis* Carr.) plantations exhibit varying levels of degradation, yet the growth trends and climate-growth relationships across different health gradients remain poorly understood.

**Methods:**

We developed tree-ring width chronologies for Chinese pine plantations representing five distinct health conditions: healthy, relatively healthy, slightly declining, moderately declining, and severely declining. Standard dendrochronological techniques were employed to compare growth rates, chronology statistical quality, and the sensitivity of radial growth to climate variables during both the growing and non-growing seasons.

**Results:**

The results showed that the healthy chronology exhibited a clear increasing trend in growth rate over time and relatively high statistical quality. In contrast, declining chronologies showed no evident long-term increase in growth rate and were characterized by lower chronology quality. Regarding climate responses, the strength of climate signals during the growing season decreased progressively with increasing decline severity. While healthy trees displayed strong positive correlations with climate variables, these relationships weakened and shifted toward negative associations in the moderately and severely declining stages. Similarly, positive climate signals in the non-growing season declined markedly along the health gradient, weakening substantially in the severely declining stage.

**Discussion:**

These findings deepen our understanding of growth decline and its environmental drivers in the Loess Plateau. The health-dependent sensitivity shifts identify a critical window for proactive intervention. Our study suggests that early detection and timely density regulation, such as thinning during mild-to-moderate decline, are essential. Furthermore, management strategies should prioritize conserving non-growing-season water—especially spring moisture—to mitigate the risk of severe forest decline and support sustainable ecological restoration.

## 1 Introduction

Chinese pine (*Pinus tabulaeformis* Carr.) is an endemic conifer in China and one of the principal zonal forest-forming species on the Loess Plateau (Li et al., 2016). Owing to its high tolerance to cold, drought, and nutrient-poor substrates, it has become the predominant plantation type for afforestation in the semi-arid northern Loess Plateau (Qin et al., 2017). In this region, Chinese pine plantations are widely distributed and play a crucial role in soil conservation and the mitigation of runoff-driven erosion (Zhang et al., 2018). Long-term monitoring in the Huanglong Mountains indicates that, compared with croplands (or recently harvested sites), Chinese pine plantations reduce surface runoff by 84.5%–88.0% and decrease soil erosion (sediment yield) by up to 99.9% (Du et al., 2003). In addition, these plantations exhibit pronounced rainfall-interception effects: mean canopy interception reaches 25.1%, stemflow accounts for 3.3% of gross precipitation, and the litter layer intercepts 11.6%, collectively weakening effective rainfall intensity and thereby reducing the potential for runoff generation (Wu et al., 1998). Chinese pine plantations also improve soil physicochemical properties. For example, soil bulk density of Chinese pine plantations is 8.6% lower than in adjacent croplands, while soil organic matter, total nitrogen, and cation exchange capacity are higher by 130.1%, 84.2%, and 53.2%, respectively (Kang et al., 2011). However, revegetation on the Loess Plateau has approached the regional limit of water-resource carrying capacity (Feng et al., 2013), and Chinese pine plantations in the Loess Plateau (LP), especially for the northern part of LP, have experienced marked growth decline and tree mortality due to soil desiccation and deterioration of microhabitat conditions (Wang et al., 2004; Shen et al., 2024). These processes have severely threaten growth health and the long-term sustainability of their ecological functions for Chine pine plantations in the Loess Plateau (Wang et al., 2005; Zhao et al., 2021).

Tree-ring archives have been widely used to investigate forest growth responses and adaptations to climate change because they provide broad spatial coverage, precise cross-dating, annual resolution, long time series, and high sensitivity to climatic variability (Cook et al., 2010; Zhang and Fang, 2020). Recently, increasing attention has been paid to tree-ring–based studies of Chinese pine forests on the Loess Plateau, particularly for elucidating historical climate variability and characterizing growth dynamics and climate sensitivity of Chinese pine forests (Liu R. et al., 2019, 2020). Using ring-width, earlywood width, wood density, and stable-isotope parameters from Chinese pine forests, numerous studies have reconstructed centennial-scale variations in temperature, precipitation, and drought indices for this region (Li et al., 2007; Shi et al., 2008; Chen et al., 2014; Hou et al., 2024). Climate sensitivity of Chinese pine radial growth exhibits pronounced spatial heterogeneity across the Loess Plateau: temperature sensitivity tends to strengthen along increasing precipitation gradients, whereas moisture limitation on tree growth is particularly evident in the northwestern Loess Plateau (Fang et al., 2012; Liang et al., 2025). In addition, micro-site conditions such as elevation and slope aspect, by modulating the local water–energy balance, can further influence the spatial differentiation of climate–growth relationships in Chinese pine forests (Li et al., 2017; Liu C. et al., 2025). Overall, tree-ring studies of Chinese pine forests on the Loess Plateau have predominantly focused on paleoclimate reconstructions and spatial heterogeneity in climate responses (Cai et al., 2015; Liu R. et al., 2019), whereas comparatively little attention has been given to using tree rings to examine growth-decline processes and associated shifts in climate sensitivity in Chinese pine plantations in this region (Wang et al., 2005; Zhao et al., 2021).

This study was conducted in the Longtan catchment, a representative area of Chinese pine plantations in the northern Loess Plateau. Under a common regional climate setting and broadly comparable site conditions, we developed tree-ring width chronologies for Chinese pine plantations spanning five health conditions (healthy, relatively healthy, slightly declining, moderately declining, and severely declining). We aimed to (i) quantify differences in chronology statistics (i.e., chronology quality) and long-term radial-growth trends among health classes, and (ii) assess how climate sensitivity of radial growth shifts and potentially transitions during the process of growth decline. This study will benefit the mechanistic understanding of growth decline and its environmental drivers for Chinese pine plantations on the Loess Plateau, and provide a scientific basis for ecological restoration and sustainable management of degraded Chinese pine plantations in this region.

## 2 Material and methods

### 2.1 Study region

**Figure 1:**
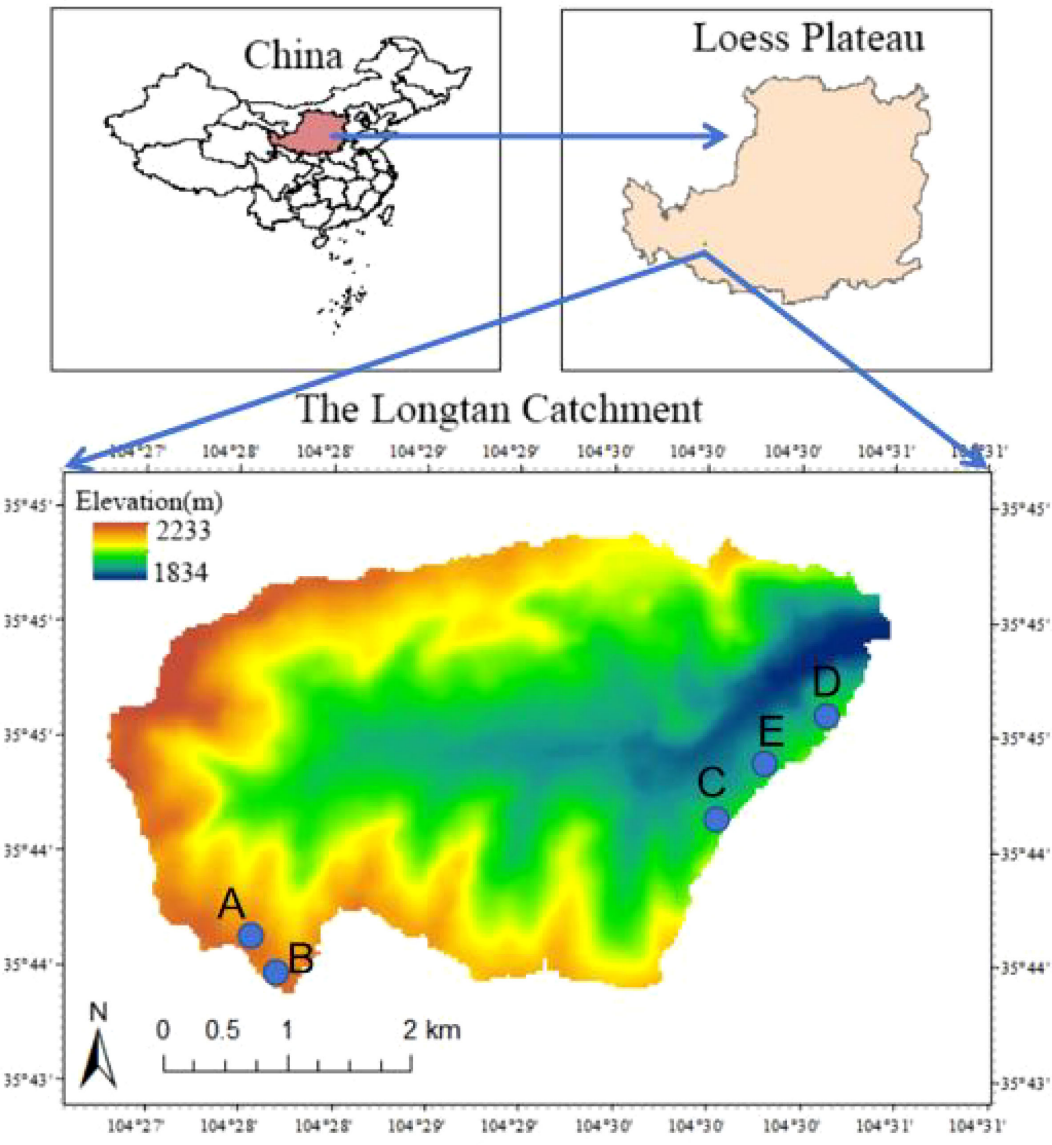
Locations of tree-ring sample sites of Chinese pine forests in the Longtan Catchment of the Loess Plateau, China. **(A–E)** indicated sample sites for very healthy, healthy, mild decline, moderate decline and severe decline Chinese pine forests, respectively.

The Longtan catchment is located in Dingxi City, Gansu Province, China (104°27′–104°32′ E, 35°43′–35°46′ N). It covers ~16.1 km² and spans an elevation range of 1840–2260 m, representing a typical loess hilly–gully landscape (**Figure 1**) (Yang et al., 2018). The region lies within the steppe zone and is dominated by Huangmian (loessial) soils, which are relatively homogeneous but characterized by a loose structure, high sand content, low fertility, and high susceptibility to erosion (Yang et al., 2020). The climate is semi-arid, with a mean annual temperature of 6.8 °C and a long-term mean annual precipitation of ~386 mm. Precipitation exhibits strong seasonality and occurs predominantly as intense rainfall events during July–September, whereas potential evaporation is high (1439–1649 mm) (Wang et al., 2019). Vegetation types within the catchment include natural grassland, shrubland, woodland, sown grassland, and cropland, forming a restoration mosaic that integrates planted vegetation with soil- and water-conservation engineering measures (Yu et al., 2018). Natural vegetation is dominated by perennial herbs, including *Stipa bungeana*, *Leymus secalinus*, *Stipa grandis*, *Heteropappus altaicus*, and *Thymus mongolicus*. Planted vegetation is mainly composed of *Caragana korshinskii*, *Platycladus orientalis*, *Armeniaca sibirica*, *Pinus tabulaeformis*, and *Medicago sativa* (Zhang et al., 2022).

### 2.2 Sample collection and chronology building

**Figure 2:**
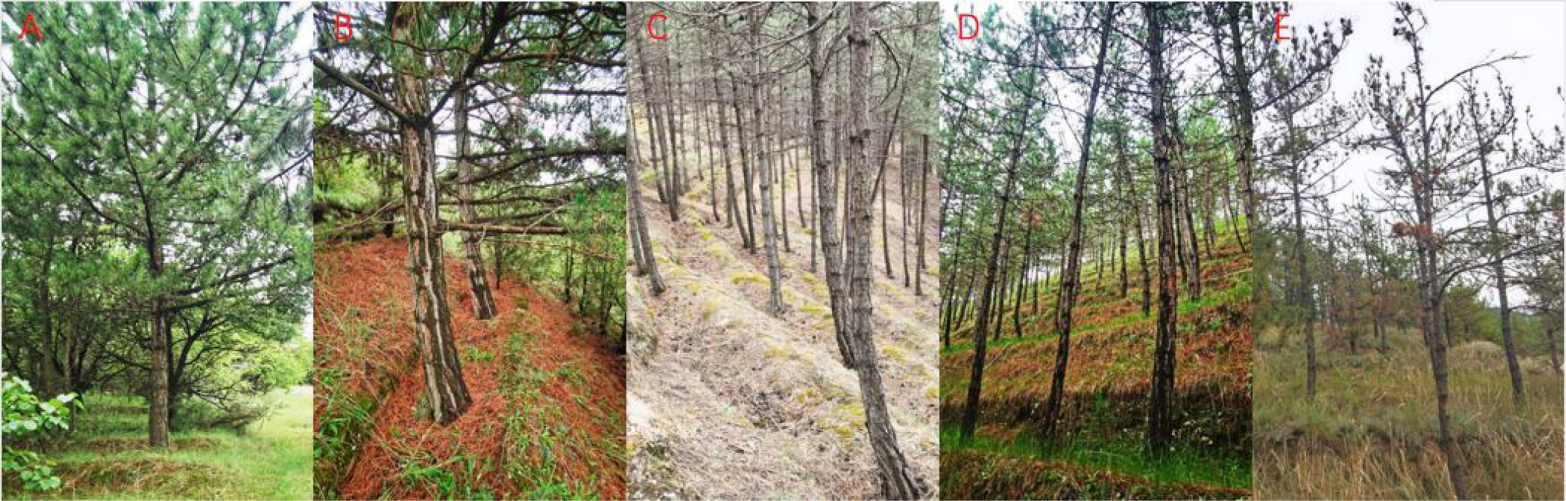
Stand-level physiognomic characteristics of Chinese pine forests in the Longtan Catchment of the Loess Plateau, China. **(A–E)** indicate the appearance of healthy, relatively healthy, slightly declining, moderately declining, and severely declining Chinese pine plantations from stand level.

In the Longtan catchment of the Loess Plateau, all tree-ring sampling sites were located on ridge and mound hilltops (i.e., upper-slope positions) to ensure broadly comparable site conditions among sites (similar elevation, slope angle, and aspect) (**Figure 1**). Sampling sites were established in representative Chinese pine plantation stands, where increment cores were collected from plantations spanning five health conditions (healthy, relatively healthy, slightly declining, moderately declining, and severely declining). Stand health classes were determined mainly based on crown dieback rate and tree appearance (**Figure 2**) (Zhu and Li, 2017). Healthy stands showed no apparent crown dieback, and trees had a diameter at breast height (DBH) of ~50 cm, with a well-developed crown and few branches on the main stem. Relatively healthy stands exhibited ~10% crown dieback, a DBH of ~40 cm, and relatively well-developed crowns with few branches on the stem. Slightly declining stands showed ~30% crown dieback, a DBH of ~30 cm, and smaller crowns with a small number of branches on the stem. Moderately declining stands exhibited ~50% crown dieback, a DBH of ~25 cm, and small crowns with more frequent branching along the stem. Severely declining stands showed ~80% crown dieback, a DBH < 20 cm, and dense, disordered branching, displaying a pronounced “old-man” (stunted, over-branched) crown form.

Tree-ring cores were extracted at breast height (~1.3 m) using an increment borer with an inner diameter of 5.15 mm. Depending on local site conditions during sampling, the coring height and direction varied slightly among trees. Each core was placed in a plastic tube, labeled, and sealed for storage (Fritts, 1966). To maximize the number of sampled individuals over a broader spatial extent under a comparable sampling effort—thereby reducing potential biases associated with inter-individual variability in chronology development (Fritts, 2001)—we sampled 60 independent trees for each health class. One core was taken from each tree, yielding 60 cores per health class and a total of 240 tree-ring cores for the entire study.

After collection, all cores were processed in the laboratory following standard procedures recommended by the Laboratory of Tree-Ring Research, University of Arizona (Stokes and Smiley, 1968). Cores were air-dried, mounted with white glue, and progressively sanded and polished using 240-, 400-, 600-, 800-, and 1000-grit sandpaper until annual ring boundaries were clearly visible and suitable for dendrochronological analysis. Ring widths were then measured to a precision of 0.001 mm using a LINTAB measuring system (Rinntech, Germany). Cross-dating and measurement accuracy were assessed with COFECHA (Grissino-Mayer, 2001). Cores showing irregular growth, decay, or breakage, and those failing to reach the 95% confidence level in correlation with the master chronology, were excluded. Consequently, 42, 43, 40, 40, and 41 cores were retained for the healthy, relatively healthy, slightly declining, moderately declining, and severely declining classes, respectively (**Table 1**). Specifically, individual ring-width series were standardized in ARSTAN by fitting negative exponential curves, and when this function did not adequately remove age-related trends, smoothing splines were applied as appropriate. This approach effectively minimized non-climatic growth trends while preserving interannual climatic signals, such as age/size effects, endogenous growth patterns, and disturbance-related release or suppression driven by inter-tree competition (Cook and Kairiukstis, 1990). Detrended series were then combined to produce standard ring-width chronologies using a biweight robust mean (Cook and Kairiukstis, 1990).

**Table 1:** Site information and statistics for standard tree-ring chronologies of different healthy classes for Chinese pine forests in the Longtan Catchment of the Loess Plateau, China.

Type	Healthy class	Location	Elevation(m)	Time length	Age	Samples	MS	SD	AC1	Rbar	SNR	EPS
Tree ring	A (healthy)	104.58°E, 35.75°N	1950	1981–2022	42	50	0.356	0.812	0.527	0.562	64.2	0.985
Tree ring	B (relatively healthy)	104.61°E, 35.81°N	2150	1980–2022	43	39	0.348	0.428	0.436	0.545	45.562	0.979
Tree ring	C (mild decline)	104.54°E, 35.79°N	2080	1983–2022	40	40	0.341	0.383	0.331	0.423	29.349	0.967
Tree ring	D (moderate decline)	104.77°E, 35.86°N	1990	1983–2022	40	43	0.344	0.374	0.278	0.401	29.494	0.967
Tree ring	E (severe decline)	104.6°E, 35.85°N	2250	1982–2022	41	33	0.337	0.428	0.313	0.323	15.294	0.939
CRU grid	—	104.5°E,35.5°N	—	1950–2024	—	—	—	—	—	—	—	—
CRU grid	—	104.5°E,40°N	—	1950–2024	—	—	—	—	—	—	—	—
CRU grid	—	105°E,35.5°N	—	1950–2024	—	—	—	—	—	—	—	—
CRU grid	—	105°E,35.5°N	—	1950–2024	—	—	—	—	—	—	—	—

MS, Mean sensitivity; SD, Standard deviation; AC1, first-order autocorrelation; Rbar, Mean series inter-correlation; SNR, Signal-to-noise-ratio; EPS, Express population signal.

### 2.3 Data analysis

Because the study sites are distant from meteorological stations and differ substantially in elevation and geomorphological settings, we used gridded climate data from the CRU TS v4.09 dataset at a spatial resolution of 0.5°(approximately 25 km) (Harris et al., 2014) to better represent watershed-scale climate variability across the loess hilly–gully terrain. We extracted monthly climate series for 1950–2024 from the four CRU grid cells closest to the sampling locations (**Figure 1**; **Table 1**). The climate variables included mean temperature, maximum temperature, minimum temperature, precipitation, and the Standardized Precipitation Evapotranspiration Index (SPEI), yielding five climatic predictors in total. For each variable, the final climate series was calculated as the average of the four grid-cell records. To account for potential lagged effects of antecedent climate on current-year growth (Fritts, 2001), we assessed climate–growth relationships over the period from June of the previous year to October of the current year by correlating monthly climate variables with the standardized ring-width chronologies. Standardized Precipitation Evapotranspiration Index (SPEI) is a multi-scalar drought index based on the standardized climatic water balance (precipitation minus potential evapotranspiration), and it integrates the combined effects of temperature-driven evaporative demand and precipitation variability on drought severity and its spatiotemporal dynamics (Vicente-Serrano et al., 2010). SPEI has been widely applied in dendroclimatological and ecological studies (Cook et al., 2010; Liu et al., 2020). Correlation analyses between tree-ring and climate variables were conducted using DendroClim2002 (Biondi and Waikul, 2004). In addition, all climatic variables in this study were calculated at a 3-month timescale. The 3-month scale were selected because this timescale aligns with the integrative nature of tree-ring growth, which reflects cumulative moisture conditions rather than short-term climatic fluctuations (Fritts, 1966; Biondi and Waikul, 2004).

## 3 Results

### 3.1 Chronology statistics

All commonly used tree-ring statistic parameters of the resulted chronologies (**Table 1**)—including mean sensitivity (MS), standard deviation (SD), first-order autocorrelation (AC1), mean series inter-correlation (Rbar), signal-to-noise ratio (SNR), and expressed population signal (EPS)—showed a clear decreasing trend along the health-to-decline gradient. Specifically, MS, SD, AC1, Rbar, SNR, and EPS decreased from 0.356, 0.812, 0.527, 0.562, 64.2, and 0.985 in the healthy chronology to 0.337, 0.428, 0.313, 0.323, 15.294, and 0.939 in the severely declining chronology, respectively. These statistics indicate that the healthy chronology has relatively higher quality and preserves a stronger common signal, implying greater sensitivity of radial growth to external environmental variability, whereas chronologies from declining stands exhibit reduced common signal strength and comparatively lower environmental sensitivity. Notably, EPS values for all health classes were well above the commonly used threshold of 0.85 (Wigley et al., 1984), suggesting that the developed chronologies adequately represent population-level growth variability in the study area and are suitable for dendroecological analyses.

### 3.2 Changing trends of climate variables and tree-ring chronologies

**Figure 3:**
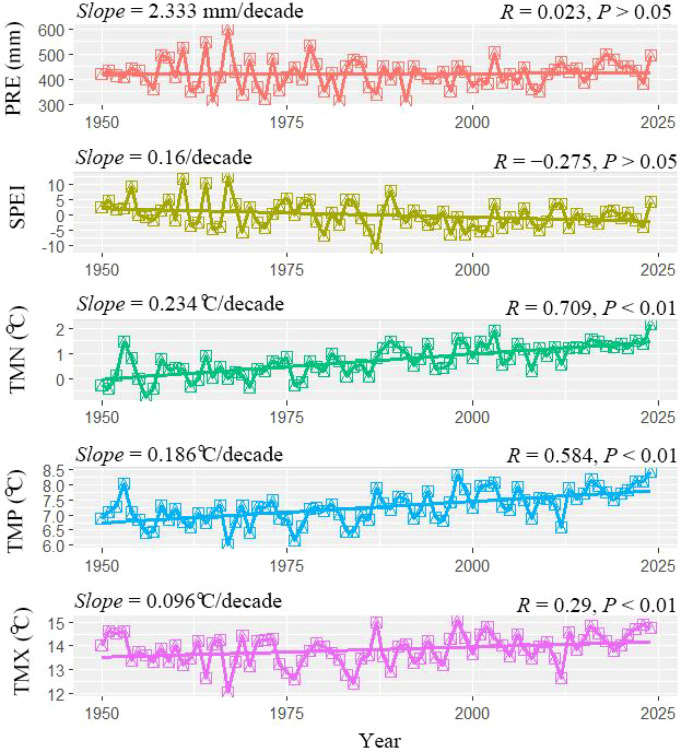
Changing trends of climatic variables during the past decades in the Longtan Catchment of the Loess Plateau, China. PRE, annual precipitation; SPEI, annual Standardized Precipitation Evapotranspiration Index; TMP, annual mean Temperature; TMX, annual maximum Temperature; TMN, annual minimum Temperature; Slope, decadal changing rate; *R*, correlation coefficient.

Based on CRU gridded climate data extracted for the location of the sampling sites (**Figure 3**), all temperature indices exhibited significant increasing trends over 1950–2024. The most pronounced warming occurred in minimum temperature (*Slope* = +0.234 °C/decade, *R* = 0.709, *P* < 0.01), followed by mean temperature (*Slope* = +0.186 °C/decade, *R* = 0.584, *P* < 0.01), whereas maximum temperature showed the weakest increase (*Slope* = +0.096 °C/decade, *R* = 0.290, *P* < 0.01). In contrast, SPEI displayed a significant decreasing trend during the same period (*Slope* = −0.16/decade, *R* = −0.275, *P* < 0.01), indicating progressive drying. Precipitation showed only a slight upward tendency (*Slope* = +2.333 mm decade, *R* = 0.023, *P* > 0.01), which was not statistically significant. Overall, these results suggest that the study area has experienced an evident warm–dry tendency over recent decades, driven primarily by rapid warming.

**Figure 4:**
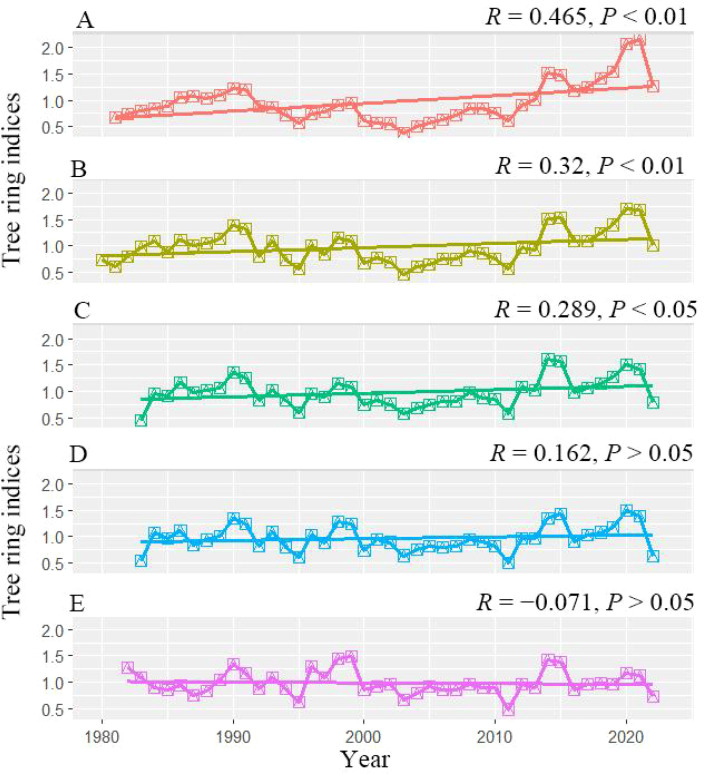
Changing trends of Chinese pine chronologies during the past decades in the Longtan Catchment of the Loess Plateau, China. **(A–E)** indicated very healthy, healthy, mild decline, moderate decline and severe decline chronology, respectively.

Based on the interannual variability of the chronologies through time (**Figure 4**), radial growth of Chinese pine in the study area declined markedly along the health-to-decline gradient. The healthy and relatively healthy classes exhibited significant increasing trends in growth rate (*R* = 0.320–0.465, *P* < 0.01). In contrast, the upward trends in the slightly and moderately declining classes were substantially weaker and not statistically significant (*R* = 0.162–0.289, *P* > 0.05). For the severely declining class, the increasing trend disappeared and shifted toward a weak decreasing tendency (*R* = −0.071, *P* > 0.05).

### 3.3 Climate-growth relationship

**Figure 5:**
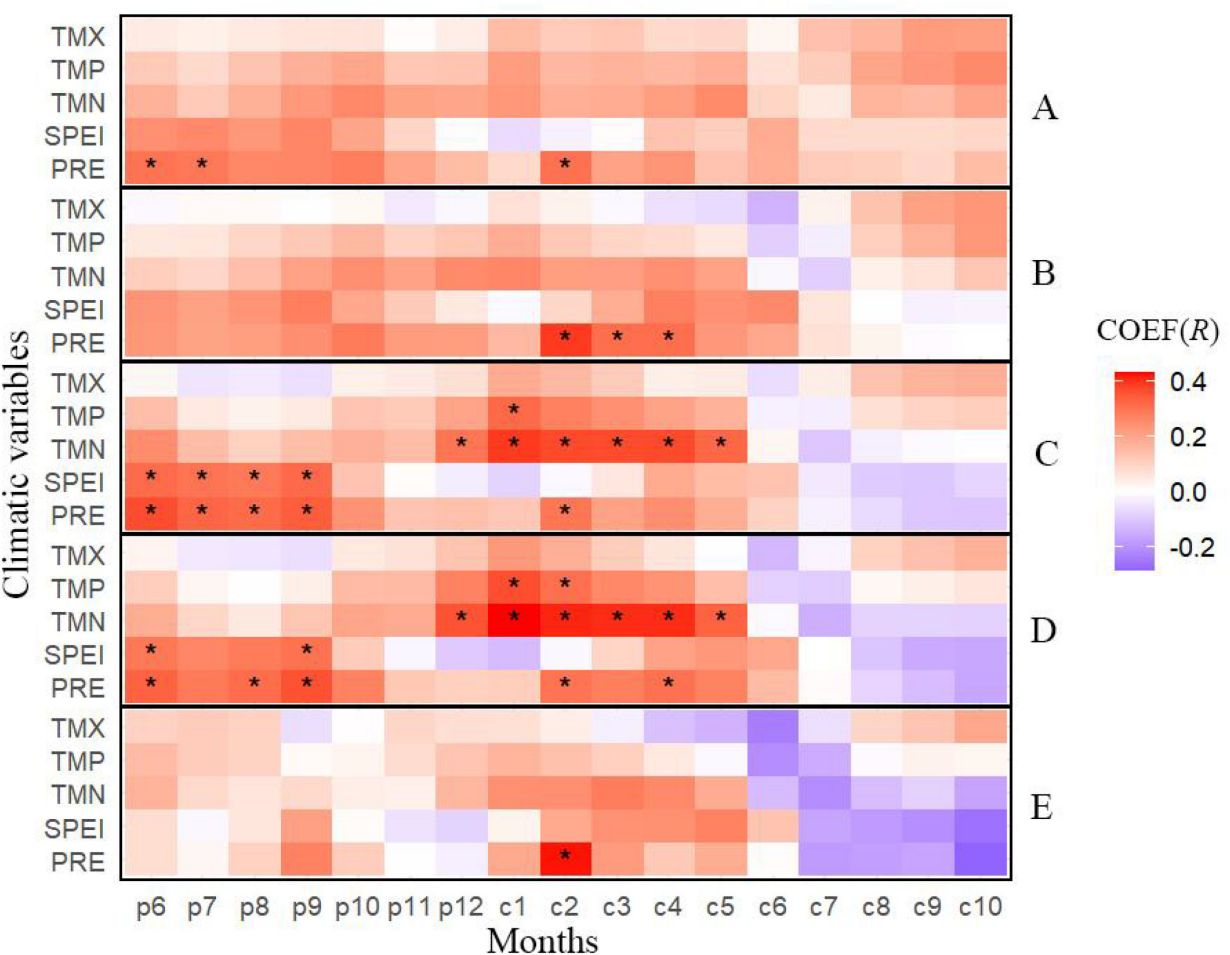
Correlations between Chinese pine chronologies in the Longtan Catchment with monthly climate factors from previous year June to current year October. TMN, Monthly Mean Minimum Temperature; TMP, Monthly Mean Temperature; TMX, Monthly Mean Maximum Temperature; PRE, Monthly Precipitation; SPEI, Standardized Precipitation Evapotranspiration Index. **(A–E)** indicated very healthy, healthy, mild decline, moderate decline and severe decline chronology, respectively.

**Figure 6:**
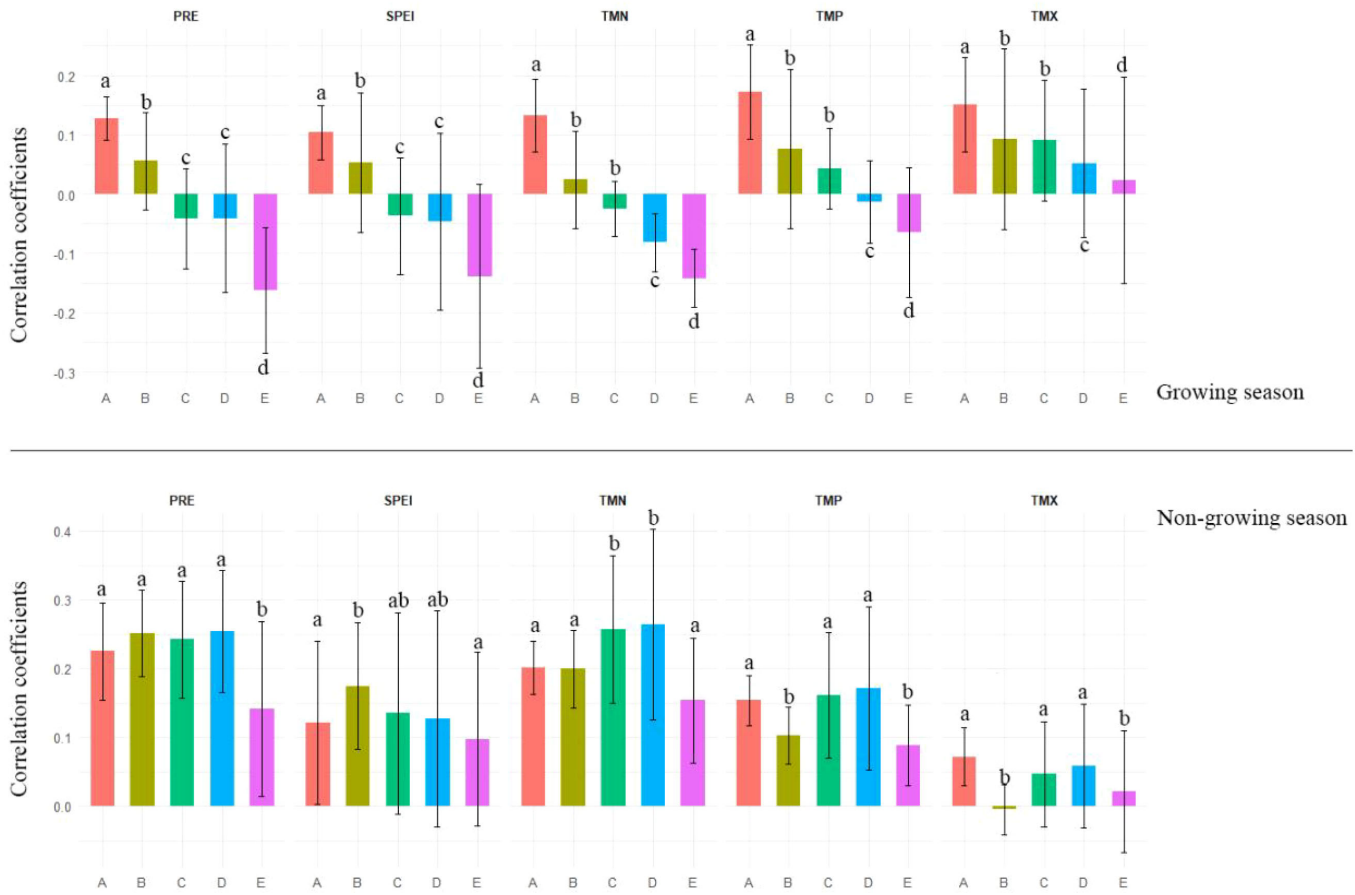
Climatic response of Chinese pine chronologies to the prominent climatic factors in the Longtan Catchment of the Loess Plateau, China. A, very healthy chronology; B, healthy chronology; C, mild decline chronology; D, moderate decline chronology; E, severe decline chronology; PRE, annual precipitation; SPEI, annual Standardized Precipitation Evapotranspiration Index; TMP, annual mean Temperature; TMX, annual maximum Temperature; TMN, annual minimum Temperature; Letters above the bars indicate the results of pairwise comparisons within each climate factor: bars sharing the same letter are not significantly different (*p* > 0.05), whereas bars with different letters differ significantly (*p* < 0.05).

**Figure 7:**
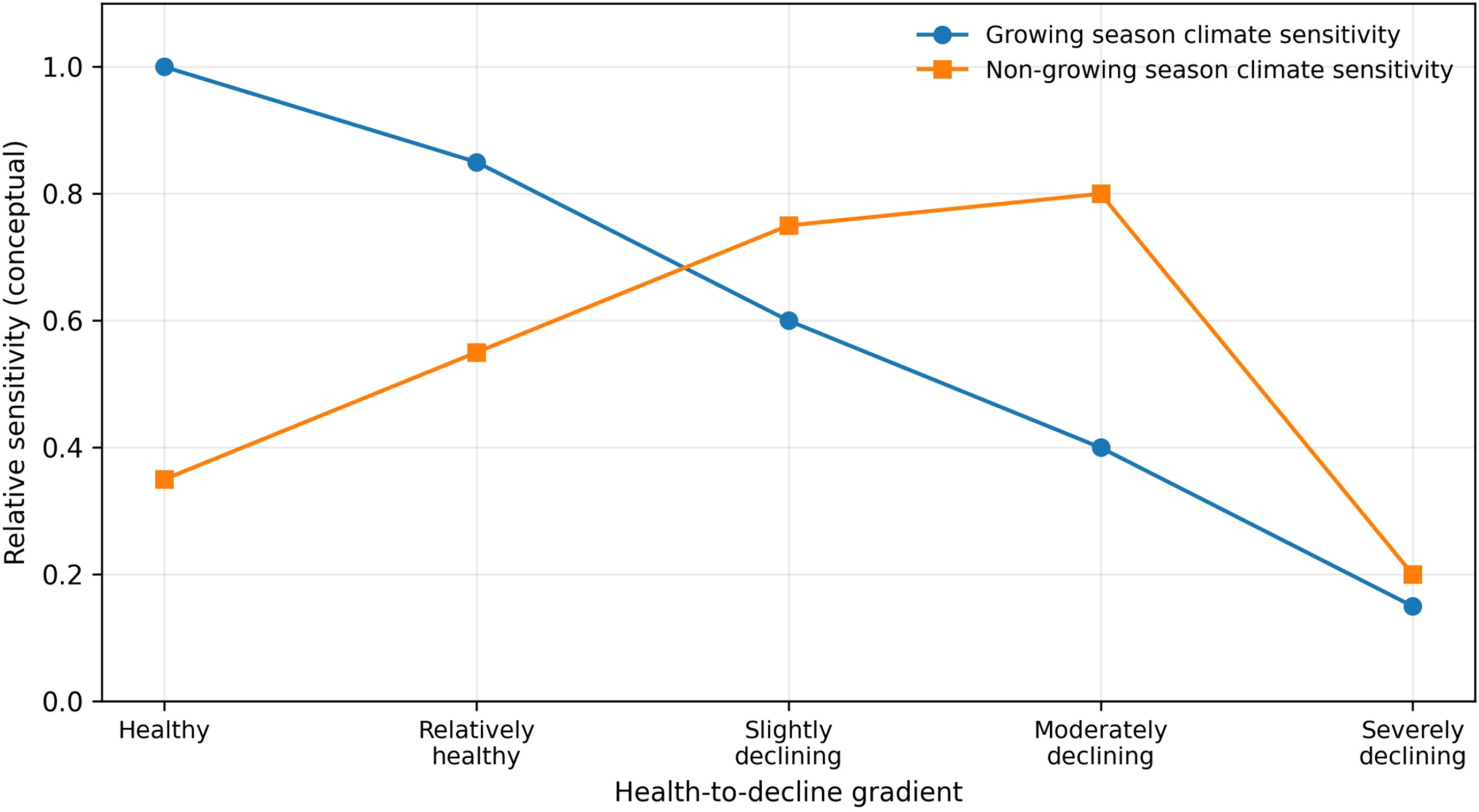
Conceptual diagram of health-dependent changes in climate sensitivity of Chinese pine along the decline gradient in the Longtan Catchment of the Loess Plateau, China. Growing-season sensitivity decreases monotonically with decline severity, whereas non-growing-season sensitivity shows a hump-shaped pattern with peak sensitivity during mild-to-moderate decline.

Based on correlation analyses between the chronologies and climatic variables (**Figures 5**–**7**), Chinese pine exhibits clear differences in climate sensitivity between the growing season (June–October of the current year) and the non-growing season (June of the previous year to May of the current year) across health classes. During the growing season, climate sensitivity declined markedly along the decline gradient. In the healthy and relatively healthy classes, ring-width indices were generally positively associated with both temperature (TMP, TMN, TMX) and moisture-related variables (PRE, SPEI). In contrast, in the mild, moderate and severe declining classes the strength of positive relationships weakened substantially (particularly for TMX), and the correlations tended to shift toward negative associations with several climate variables (PRE, SPEI, TMN, TMP).

During the non-growing season, climate sensitivity of Chinese pine chronologies exhibited a hump-shaped pattern along the decline gradient, increasing from the healthy class to the relatively healthy, slightly declining, and moderately declining classes, and then decreasing sharply to the lowest level in the severely declining class (**Figures 5**–**7**). This pattern was most pronounced for precipitation (PRE) and minimum temperature (TMN), followed by SPEI and mean temperature (TMP). In contrast, responses to maximum temperature (TMX) were weakest overall, and the corresponding trend was less distinct.

## 4 Discussion

### 4.1 Growth decline characteristics of Chinese pine plantations

Extreme drought conditions associated with global warming have already triggered widespread forest growth decline and tree mortality, thereby exerting substantial negative impacts on the ecosystem carbon sink function of forests at regional scales (Choat et al., 2012; McDowell et al., 2022). Tree-ring evidence from many regions worldwide indicates that tree growth rates have declined markedly in recent decades, and forest growth has decreased to some extent due to the adverse effects of drought stress even in tropical rainforests (Briffa et al., 1998; Cabon et al., 2022). Against the backdrop of global climate change, the Loess Plateau—an ecologically fragile region located at the margin of the Asian monsoon domain—shows particularly pronounced vegetation responses to a “warming–drying” climate (Feng et al., 2013). Based on extensive dendrochronological datasets, interannual forest growth rates in the Loess Plateau have exhibited a certain downward trend (Chen et al., 2014; Liu et al., 2020).

In this study, although the radial growth rate of Chinese pine plantations in the Longtan watershed decreased progressively along the decline gradient, the decline stage was mainly characterized by a slowdown in the rate of growth increase rather than a distinct growth downturn (**Figure 4**). Previous studies have reported clear recent growth declines in other typical plantation species on the Loess Plateau, such as black locust (*Robinia pseudoacacia*) and simon poplar (*Populus simonii*), where drought stress has led to pronounced reductions in radial growth rates (Yan et al., 2022; Liu Y. et al., 2025). In contrast, the growth decline signal in Chinese pine plantations within our study area appears comparatively weak. Chinese pine has strong survival advantages on the Loess Plateau under infertile, shallow, or rocky-substrate soils, and is characterized by high drought and cold tolerance (Li et al., 2016). Compared with broadleaved species that require higher nutrient and water availability, Chinese pine exhibits greater ecological resilience under intensifying drought stress (Wang et al., 2017; Hou et al., 2024), and thus does not display the pronounced growth decline commonly observed in many broadleaved species (Zhang et al., 2020; Li et al., 2025).

### 4.2 Climatic sensitivity of radial growth for Chinese pine plantations along the health-to-decline gradient

This study found that the climate-response sensitivity of Chinese pine plantations in the Longtan catchment on the Loess Plateau exhibited clear variation along the growth-decline gradient (**Figures 5**–**7**). During the growing season (June–October of the current year), climate-response sensitivity showed a decreasing trend with increasing decline severity. The growing season represents the most vigorous period of radial growth and the phase when physiological metabolism imposes the strongest demand for water (Cai et al., 2015; Liu Y. et al., 2019). At the healthy stage, Chinese pine possesses relatively strong hydraulic regulation capacity and carbon reserves, enabling effective use of the coupled conditions of growing-season precipitation and moderate temperatures (Chen et al., 2014). This promotes xylem tracheid division and enlargement via enhanced photosynthetic efficiency, thereby producing wider annual rings (Castagneri et al., 2020), which is expressed as positive correlations with climate variables. As trees enter decline, deteriorating site conditions drive multiple physiological functions toward, or beyond, tolerance thresholds, resulting in a loss of the capacity to regulate responses to climatic fluctuations (Du et al., 2003; Fonti and Garcia-Gonzalez, 2008) and manifesting as negative correlations with climate variables. The pronounced shift in climate-response sensitivity between healthy and declining trees during the growing season likely reflects the broader context of regional warming, under which intensified water stress reduces ecological resilience and forces a transition from an active adjustment strategy to a more passive stress-tolerance mode of survival (Choat et al., 2012; Anderegg et al., 2018). Interesting, healthy trees have a lower correlation with summer SPEI compared to declining ones (**Figure 5**). We interpret the weaker summer SPEI sensitivity in healthy trees as a buffering effect, likely reflecting stronger water-use regulation and access to more stable water sources (e.g., deeper/more effective roots and better stomatal control) (Choat et al., 2018). In contrast, declining trees may have impaired root function and reduced regulation, increasing their sensitivity to short-term moisture deficits (Babst et al., 2019).

In addition, this study showed that, during the non-growing season, the climate-response sensitivity of Chinese pine first increased and then decreased along the decline gradient. In the healthy stage, strong intrinsic regulation and high stress resistance reduce dependence on subtle non-growing-season climatic variability, leading to comparatively weak relationships with climate factors (Allen et al., 2010; Choat et al., 2012). Trees in mild-to-moderate decline often experience root impairment and/or intensified competition, making growth highly dependent on non-growing-season water inputs-particularly snowmelt and precipitation in spring and early spring (Anderegg et al., 2018; Babst et al., 2019). This increases sensitivity to environmental conditions and strengthens correlations with climatic variables. By contrast, under severe decline, irreversible physical damage to the hydraulic transport system combined with depletion of non-structural carbohydrate reserves reduces the ability to perceive and respond to external climatic signals (Adams et al., 2017; Choat et al., 2018), resulting in the weakest relationships with climate variables. Notably, compared with healthy trees, declining trees are more susceptible to the influence of climatic conditions in the previous June on their current-year growth (**Figure 5**). Chronic stress may reduce photosynthetic carbon gain and deplete non-structural carbohydrate reserves in the declining trees, and this could limit subsequent cambial activity and thereby strengthen lagged climate-growth relationships (Fritts, 1966; Allen et al., 2010).

### 4.3 Pathways to structural renovation and functional enhancement of degraded Chinese pine plantations on the Loess Plateau

This study identified growth-decline characteristics and climate-sensitivity patterns of planted Chinese pine stands in the Longtan catchment on the Loess Plateau under a “warming–drying” background, providing practical ecological implications for structural improvement, functional enhancement, and sustainable management of regional plantations (Liu et al., 2020). Our results indicate that, in drought-prone habitats, Chinese pine exhibits higher ecological resilience than broadleaved species (e.g., black locust and simon poplar). However, during the moderate-decline stage, trees show a markedly increased dependence on non-growing-season moisture, likely associated with impaired belowground root functioning and/or intensified competition (Kang et al., 2011). To alleviate competition-driven water stress, scientifically designed thinning should be implemented to reduce stand density. Such interventions can effectively decrease inter-individual competition for limited soil water and enhance stand-level stress resistance (Jiang et al., 2021). Given the pronounced survival advantage of Chinese pine, structural renovation should capitalize on its cold and drought tolerance and promote the establishment of mixed conifer–broadleaf stands through enrichment planting with native species (Jiao et al., 2010).

We further show that the transition from healthy to declining conditions in Chinese pine is accompanied by a shift from an “active adjustment” strategy to a more “passive tolerance”. The severe-decline stage is characterized by irreversible hydraulic physical damage and depletion of carbon reserves. For stands in mild-to-moderate decline, improving surface cover (e.g., straw mulching or biological soil crusts) can reduce soil evaporation, particularly during critical non-growing-season periods such as spring and early spring, thereby safeguarding tree water balance (del Campo et al., 2020). In healthy trees, enhanced photosynthetic efficiency may promote tracheid division and the formation of wider rings, whereas decline substantially constrains carbon balance (Cabon et al., 2022). Targeted management that maintains stands in healthy or relatively healthy conditions can maximize the ecological carbon-sink function of plantations and reduce the risk of a shift from a carbon sink to a carbon source triggered by widespread mortality (Feng et al., 2013, 2016).

Notably, the climate sensitivity of Chinese pine displays systematic fluctuations along the decline gradient, offering a management-relevant temporal window and diagnostic indicators for sustainability. By leveraging the identified sensitivity-transition features in the climate response of radial growth, in combination with remote-sensing monitoring (e.g., non-photosynthetic vegetation index, NPV, or resilience-related metrics), an early-warning system for forest decline can be developed (Chen et al., 2021). Before trees enter the irreversible severe decline stage, stands showing anomalously elevated sensitivity during mild-to-moderate decline should be detected and management interventions applied in a timely manner. For healthy and relatively healthy stands, close-to-nature management is recommended to maintain strong self-regulatory capacity (Bachofen et al., 2024). For stands in mild-to-moderate decline, priority should be given to securing non-growing-season water supply (e.g., snow management and/or supplemental spring irrigation), because trees at this stage may be highly dependent on spring moisture inputs (Ciruzzi and Loheide, 2021). When stands transition toward severe decline, physiological functioning may approach threshold limits and the capacity to record diagnostic signals becomes weak; stand renewal or replacement should then be considered, selecting more drought-tolerant genotypes or species to support long-term sustainability of forest resources (Fu et al., 2022, 2023). Overall, the growth-response patterns revealed here provide a theoretical basis for shifting management of Loess Plateau plantations from passive response toward proactive early warning and precision intervention.

## 5 Conclusion

• Long-term growth trajectories and chronology quality of Chinese pine plantations deteriorated progressively from healthy to severe decline classes, indicating a systematic weakening of stand-scale growth coherence in the semi-arid Loess Plateau, China.

• Sensitivity to growing-season climate weakened with increasing decline severity and tended to shift from positive to negative associations with key moisture- and temperature-related variables.

• Non-growing-season climate signals followed a hump-shaped pattern, with peak sensitivity during mild-to-moderate decline, consistent with increasing dependence on antecedent moisture as hydraulic functioning degrades.

• Management should prioritize early detection and timely density regulation (e.g., thinning) before stands enter severe decline, and strengthen measures that conserve non-growing-season water (especially spring moisture) to sustain plantation functions under ongoing warming and drying climate conditions.

## Data Availability

The raw data supporting the conclusions of this article will be made available by the authors, without undue reservation.
